# Supplementary material for: Development and Optimization of a Germination Assay and Long-Term Storage for Cannabis sativa Pollen
Source: Plants (Basel). 2020 May 23;9(5):665. doi: 10.3390/plants9050665 (PMC7284845; doi:10.3390/plants9050665)
Supplement: Supplementary file 1 [file plants-09-00665-s001.zip › Supplementary Figures file.docx]

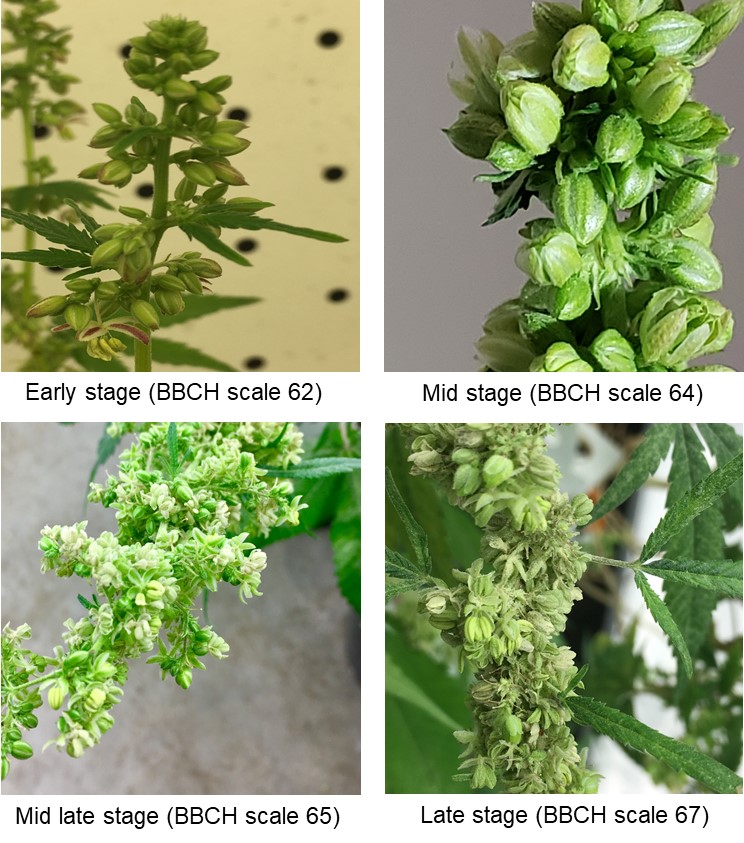


**Figure S1:** The representative photographs of the male inflorescences at various stages of flower development. The four stages of flowering were chosen according to the BBCH (Biologische Bundesantalt, Bundessortenamt and Chemische) scale adapted for cannabis (Mishchenko et al. 2017) and are listed as follows with the BBCH notation in brackets: Early (62), Mid (64), Mid-Late (65) and Late (67).

Reference: Mishchenko, S.; Mokher, J.; Laiko, I.; Burbulis, N.; Kyrychenko, H.; Dudukova, S. Phenological growth stages of hemp (Cannabis sativa L.): codification and description according to the BBCH scale. *Žemės ūkio Moksl.* **2017**, *24*.


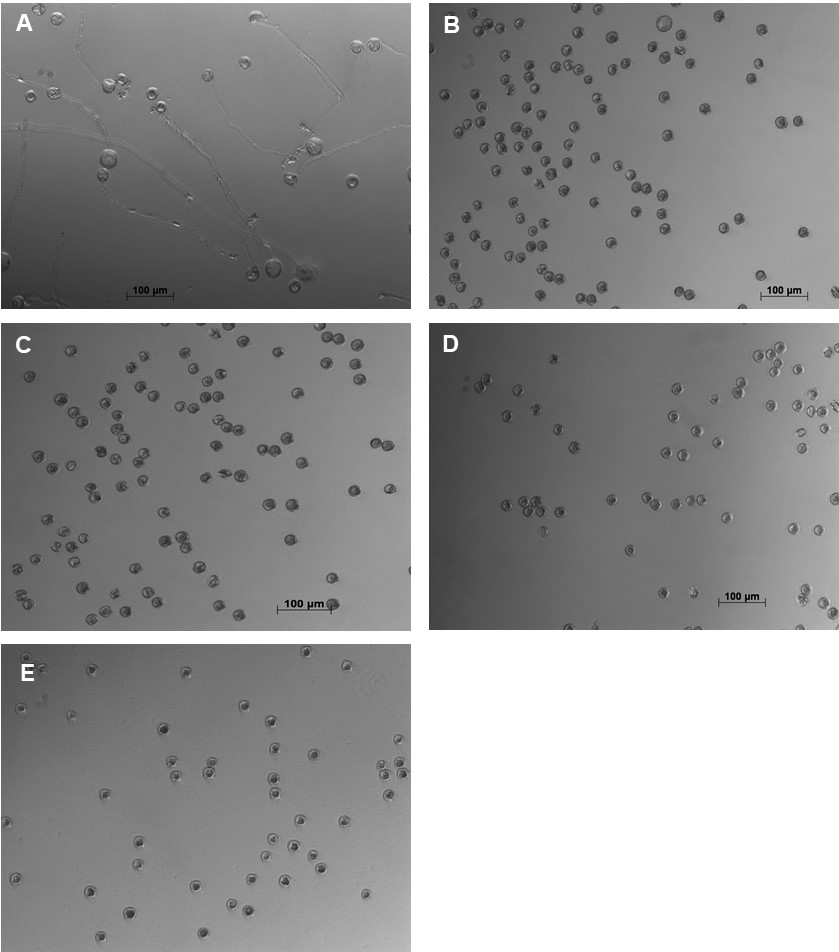


**Figure S2.** Representative photographs from pollen germination assay (PGA). (A) Control fresh pollen, (B) Pollen stored in liquid nitrogen without prior vacuum desiccation, (C) Pollen stored in liquid nitrogen with prior vacuum desiccation at 5 kPa for 40 minutes, (D) Pollen stored in liquid nitrogen with prior vacuum desiccation combined with 10% DMSO solution, and (E) Pollen stored in liquid nitrogen with prior vacuum desiccation combined with 10% Glycerol solution.


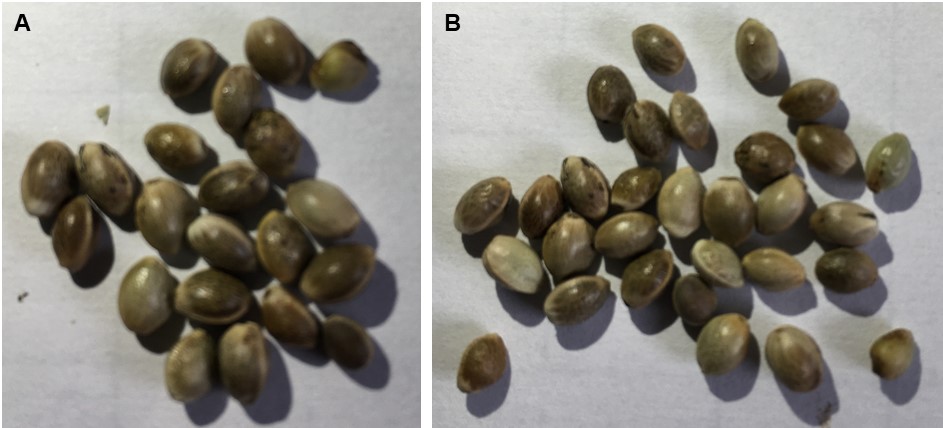


**Figure S3.** Photographs of harvested seeds from (A) flower pollinated with control fresh pollen, and (B) flower pollinated with stored pollen (desiccated pollen/ wheat flour mix stored in liquid nitrogen.
